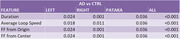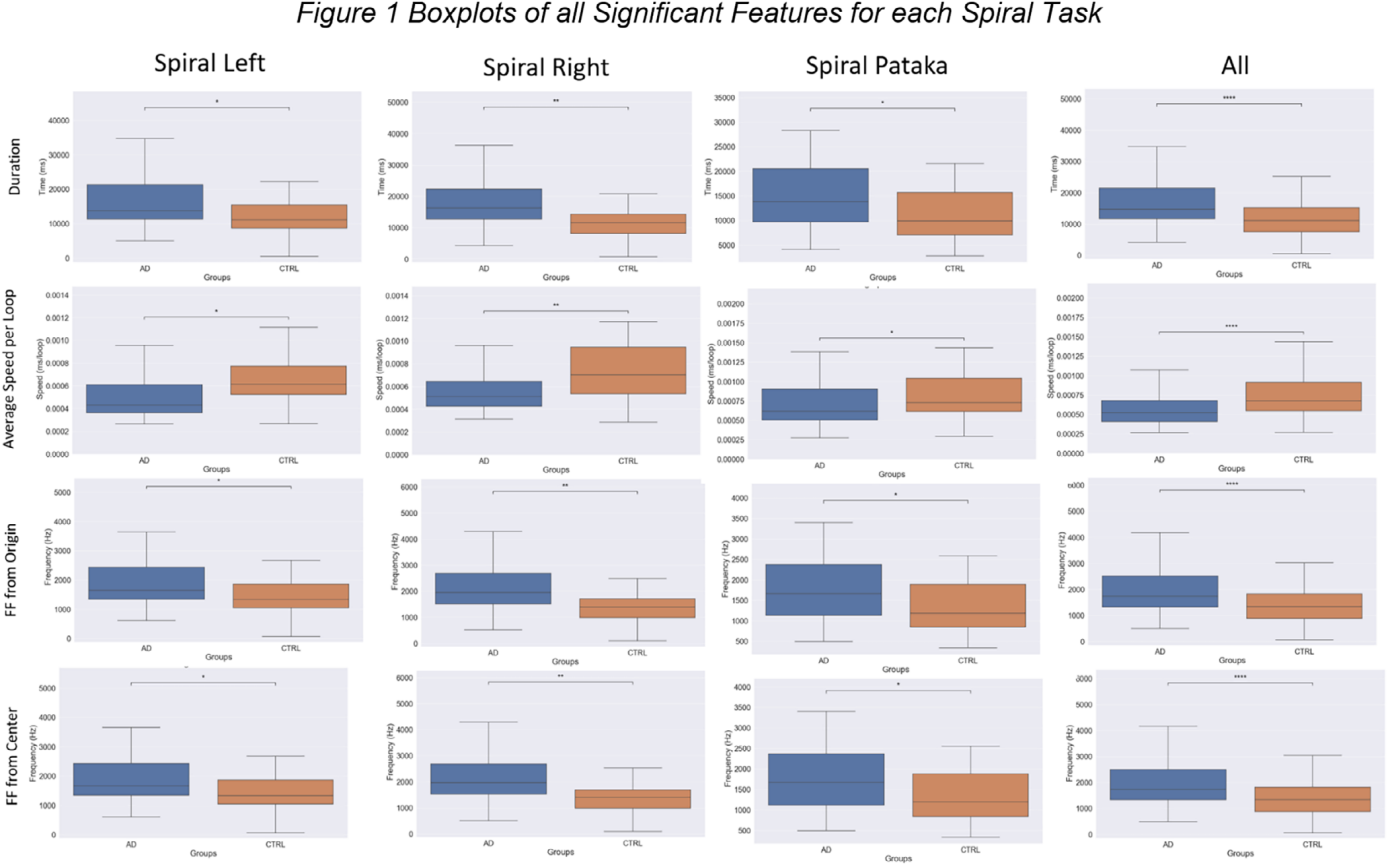# Dynamics of Handwriting for Cognitive Assessment

**DOI:** 10.1002/alz.093190

**Published:** 2025-01-09

**Authors:** Gabrielle Chavez, Thomas Thebaud, Ankur Butala, Najim Dehak, Laureano Moro‐Velazquez, Esther S Oh

**Affiliations:** ^1^ Johns Hopkins University, Baltimore, MD USA; ^2^ Johns Hopkins University School of Medicine, Baltimore, MD USA

## Abstract

**Background:**

Previous studies have shown that motor dysfunction can be an indicator of AD. We measured the observable and dynamic characteristics of spirals drawn by people with AD.

**Method:**

We instructed 23 AD and 40 healthy control (CTRL) participants to perform a range of handwriting tasks on a digital tablet using a pen. We instructed the participants to draw spirals in three different ways with: i) left hand; ii) right hand; iii) dominant hand while repeating the occlusive word “Pa‐ta‐ka.” From the drawing samples of 63 participants collected over multiple sessions, we computed 14 time‐independent, explainable features from the shape of the spirals and the dynamics of the drawings (duration, fundamental frequencies using Fourier transform etc.). Those features were compared between the two studied groups using Welch's t‐test with a false discovery rate (FDR) correction.

**Result:**

Right Spirals

We found there is a significant difference (p‐value<0.01) for the Duration and Fundamental Frequency (FF) with respect to Origin vs Total Number of Points, and FF with respect to Center vs Total Number of Points. Furthermore, the Average Loop Speed is significant (p‐value<0.05).

Left Spirals

We found there is a significant difference (p‐value<0.05) for all features.

Pataka Spirals

Additionally, there is a significant difference (p‐value<0.05) for AD and CTRL participants concerning the Duration, Average Loop Speed, and FF Origin and FF Center.

All Spirals

We compared all spiral samples based on only their group type, either AD or CTRL. We found that all features resulted in a significant difference (p‐value<0.01).

**Conclusion:**

Though there was no notable visual variation between AD and CTRL spirals, we did find there were significant differences among dynamical characteristics like duration, loop speed, and frequencies. We can conclude that AD participants tend to take longer drawing spirals which result in lower Average Speed per Loop than their CTRL counterparts. Additionally, the FF extracted from both groups indicate that higher frequencies are found in AD participants.